# ABCD3 is a prognostic biomarker for glioma and associated with immune infiltration: A study based on oncolysis of gliomas

**DOI:** 10.3389/fcimb.2022.956801

**Published:** 2022-07-25

**Authors:** Jinchuan Li, Yi Zhang, Zhizhao Qu, Rui Ding, Xiaofeng Yin

**Affiliations:** ^1^ Department of Neurosurgery, Second Hospital of Shanxi Medical University, Taiyuan, China; ^2^ Department of Neurosurgery, First Hospital of Shanxi Medical University, Taiyuan, China

**Keywords:** glioma, ABCD3, biomarker, oncolytic virus, immune infiltrates

## Abstract

**Background:**

Gliomas are the most lethal primary brain tumors and are still a major therapeutic challenge. Oncolytic virus therapy is a novel and effective means for glioma. However, little is known about gene expression changes during this process and their biological functions on glioma clinical characteristics and immunity.

**Methods:**

The RNA-seq data after oncolytic virus EV-A71 infection on glioma cells were analyzed to screen significantly downregulated genes. Once ABCD3 was selected, The Cancer Genome Atlas (TCGA), Chinese Glioma Genome Atlas (CGGA), Genotype-Tissue Expression (GTEx), Clinical Proteomic Tumor Analysis Consortium (CPTAC), and Human Protein Atlas (HPA) data were used to analyze the relationship between ABCD3 expression and clinical characteristics in glioma. We also evaluated the influence of ABCD3 on the survival of glioma patients. CIBERSORT and Tumor Immune Estimation Resource (TIMER) were also used to investigate the correlation between ABCD3 and cancer immune infiltrates. Gene set enrichment analysis (GSEA) was performed to functionally annotate the potential functions or signaling pathways related to ABCD3 expression.

**Results:**

ABCD3 was among the top 5 downregulated genes in glioma cells after oncolytic virus EV-A71 infection and was significantly enriched in several GO categories. Both the mRNA and protein expression levels of ABCD3 were upregulated in glioma samples and associated with the prognosis and grades of glioma patients. The Kaplan–Meier (K-M) curve analysis revealed that patients with high ABCD3 expression had shorter disease-specific survival (DSS) and overall survival (OS) than those with low ABCD3 expression. Moreover, ABCD3 expression could affect the immune infiltration levels and diverse immune marker sets in glioma. A positive correlation was found between ABCD3 and macrophages and active dendritic cells in the microenvironment of both the GBM and LGG. Gene sets including the plk1 pathway, tyrobp causal network, ir-damage and cellular response, and interleukin-10 signaling showed significant differential enrichment in the high ABCD3 expression phenotype.

**Conclusion:**

Our results suggested that ABCD3 could be a potential biomarker for glioma prognosis and immunotherapy response and also further enriched the theoretical and molecular mechanisms of oncolytic virus treatment for malignant gliomas.

## Introduction

Gliomas are the most common form of primary brain tumors. They are generally divided into glioblastoma multiforme (GBM) and low-grade glioma (LGG) (Alifieris and Trafalis, 2015; Chen et al., 2017; Hu et al., 2021b). GBM is classified by the WHO as a grade IV tumor that is highly invasive and rapidly growing and has massive metastases (Hu et al., 2021a). Despite the currently available strategies of surgical resection, chemotherapy, and irradiation, the median survival for patients is only 12–15 months (Batash et al., 2017; Witthayanuwat et al., 2018). LGG (grades II and III) is a more inert precursor to glioblastoma and the prognosis is relatively encouraging, despite the possibility of evolving into a more aggressive GBM (Wang and Mehta, 2019). Usually, the disease remains unchanged for a long period of time. However, patients have few effective treatment options, and the prognosis for these patients today is poor (Alexander and Cloughesy, 2017). So, the search for new biomarkers in glioma patients is particularly urgent in order to provide a highly accurate prediction of patient survival and/or response to individualized treatment.

Several studies have confirmed the safe and effective use of oncolytic viruses in the treatment of glioma (Foreman et al., 2017; Suryawanshi and Schulze, 2021). Oncolytic virus therapy is an immunotherapy treatment that is characterized by virus-specific infection of glioma cells and apoptosis induction through the release of viral progeny (Zeng et al., 2021). The oncolytic virus treatment on glioma often results in the downregulation of genes that promote glioma malignancy, as well as changes in molecules associated with the immune response (Rius-Rocabert et al., 2020). A recent study shows that enterovirus A71 (EV-A71) has potent oncolytic activity in the treatment of glioma (Zhang et al., 2020a). Malignant glioma-derived cell lines are capable of being infected and killed by EV-A71 (Zhang et al., 2020a). An EV-A71 treatment can significantly slow down the progression of tumors in nude mice bearing glioma xenografts (Zhang et al., 2020a). Oncolytic viruses are capable of exhibiting an antitumor effect that is connected to both their intrinsic oncolytic properties and the way the immune system reacts (Hemminki et al., 2020).

In the treatment of glioma with EV-A71, we screened a significantly downregulated ATP-binding cassette (ABC) transporter subfamily D member 3 (ABCD3) by high-throughput sequencing data. Based on structural organization and amino acid homology, ABC transporters are classified into seven subfamilies (A to G) in humans (Dean et al., 2001). ABCD3, also known as the 70-kDa peroxisomal membrane protein (PMP70), is localized to the monolayer of the peroxisome, and the expression of the membrane protein increases as the peroxisome proliferates (Braiterman et al., 1998). The encoded protein is involved in the transport of fatty acids or fatty acyl coenzyme A in the peroxisome (Imanaka et al., 2000). Many studies have confirmed that ABCD3 plays an important role in the development and progression of many tumors and involves several regulatory mechanisms in tumor development (Reams et al., 2015; Zhang et al., 2020b). However, ABCD3 has hardly been evaluated in glioma.

In this study, our data were collected from the European Nucleotide Archive (ENA), The Cancer Genome Atlas (TCGA), Chinese Glioma Genome Atlas (CGGA), Genotype-Tissue Expression (GTEx), and Human Protein Atlas (HPA). Moreover, the Gene Expression Profiling Interactive Analysis (GEPIA) and R package identified the differences and correlations of ABCD3 with glioma. We evaluated the correlations between clinical characteristics and ABCD3 in glioma. Subsequently, the density of tumor-infiltrating immune cells (TIICs) in tumor microenvironments was studied using a recent metagene approach called CIBERSORT, as well as the Tumor Immune Estimate Resource (TIMER). We also examined the association between ABCD3 and infiltrating immune cells in tumors using these methods. According to our results, ABCD3 proves to be an effective diagnostic and prognostic biomarker for gliomas and to have a possible correlation with tumor immune interactions.

## Materials and methods

### Data acquisition and bioinformatics

The RNA-seq data after EV-A71 infection on glioma cells were obtained from the ENA with accession number PRJNA562271. In that study, glioma cells were infected with EV71 BrCr strain or mock infection at a multiplicity of infection (MOI) of 1 for 60 min at room temperature. The total RNA samples from CCF glioma cells were used for RNA-seq analysis by using the Illumina HiSeq™ 2000 System (Illumina, San Diego, USA). After getting the raw data, we utilized FastQC for quality testing. The differentially expressed genes (DEGs) were calculated by Gfold version 1.1.4, (Shanghai, China) and a GFOLD value ≥1 or ≤−1 was considered.

GBM and LGG patient datasets were downloaded from the TCGA, CGGA, and GTEx with ABCD3 gene expression and the corresponding clinical information data. The protein expression data were evaluated using the Human Protein Atlas and Clinical Proteomic Tumor Analysis Consortium (CPTAC) databases.

### Survival analysis

For survival analysis, we compared the overall survival (OS) and disease-specific survival (DSS). Both GBM and LGG patients were divided separately into high and low ABCD3 groups based on the mean value of TPM. Survival analysis and visual representations were executed using R version 3.4.3, including survival (for survival analysis and Cox) and survminer (for visual representations) R packages. Kaplan–Meier (K-M) curves were carried out to compare the survival time differences. *P*-values from log-rank tests were calculated, and less than 0.05 was considered statistically significant.

### ABCD3 expression and tumor-infiltrating immune cell analysis

To analyze the ABCD3 RNA sequencing expression data from tumors and normal samples in the GTEx and TCGA projects, a webserver called GEPIA was used as a standard processing pipeline. Plotting of ABCD3 expression was performed using ggplot2.

To assess the influence of ABCD3, we utilized two methods to estimate the infiltration cells. Firstly, we employed the single-sample gene set enrichment analysis (ssGSEA) using the R gene set variation analysis (GSVA) package (v1.34.0) to calculate the infiltration levels of 24 immune cells. The marker genes for these 24 immune cells come from this article (Bindea et al., 2013). To compare two groups of values, the Wilcoxon signed-rank tests or the two-sided non-parametric Wilcoxon rank-sum tests were used. The second method is TIMER (cistrome.shinyapps.io/timer), which is a web-based evaluation of the amount of tumor-infiltrating immune cells (Li et al., 2017). All GBM and LGG data were downloaded from the TCGA. The scatterplots displayed the purity-corrected partial Spearman’s rho value and statistical significance.

### Gene set enrichment analysis

To explore the potential signaling pathways and biological functions related to ABCD3 expression, we conducted gene set enrichment analysis (GSEA) based on MSigDB Collections using the clusterProfiler R package (v3.14.3). The GBM and LGG samples were sorted into high and low expression groups, respectively, according to ABCD3 expression. The different transcriptional profiles were identified and sorted by log2FC with DESeq2. Then, we ran the GSEA on these transcriptional profiles and assessed the enrichment using the normalized enrichment score (NES) as a metric of enrichment. False discovery rate (FDR) <0.25 and p.adjust value <0.05 were considered to be of statistical significance.

### Statistical analysis

Statistical analyses from the TCGA were merged and performed using R version 3.4.3. The correlations between ABCD3 expression and clinical information were examined using logistic and Cox regressions. GraphPad Prism was used to perform statistical analysis. A two-tailed *P* < 0.05 was considered statistically significant.

## Results

### ABCD3 is significantly decreased during oncolytic virus-mediated apoptosis in glioma cells

It has been reported that oncolytic viruses EV-A71 have shown oncolytic efficacy in glioma (Zhang et al., 2020a). Based on this finding, we conducted the transcriptome analysis to further analyze the impact of EV-A71 infection on glioma cells. The RNA-seq data (PRJNA562271) were obtained from CCF glioma cells after EV71 or mock infection at an MOI of 1. After getting the raw data, we utilized FastQC for quality testing. The results confirmed a high level of quality and sufficient quantity for further gene functional analysis (**Supplementary Figure 1**). Using the TPM method (the number of transcripts per million clean tags), the gene expression level was calculated and normalized by Gfold. The distribution of DEGs from EV-A71 groups was obtained relative to the mock infection.

**Figure 1:**
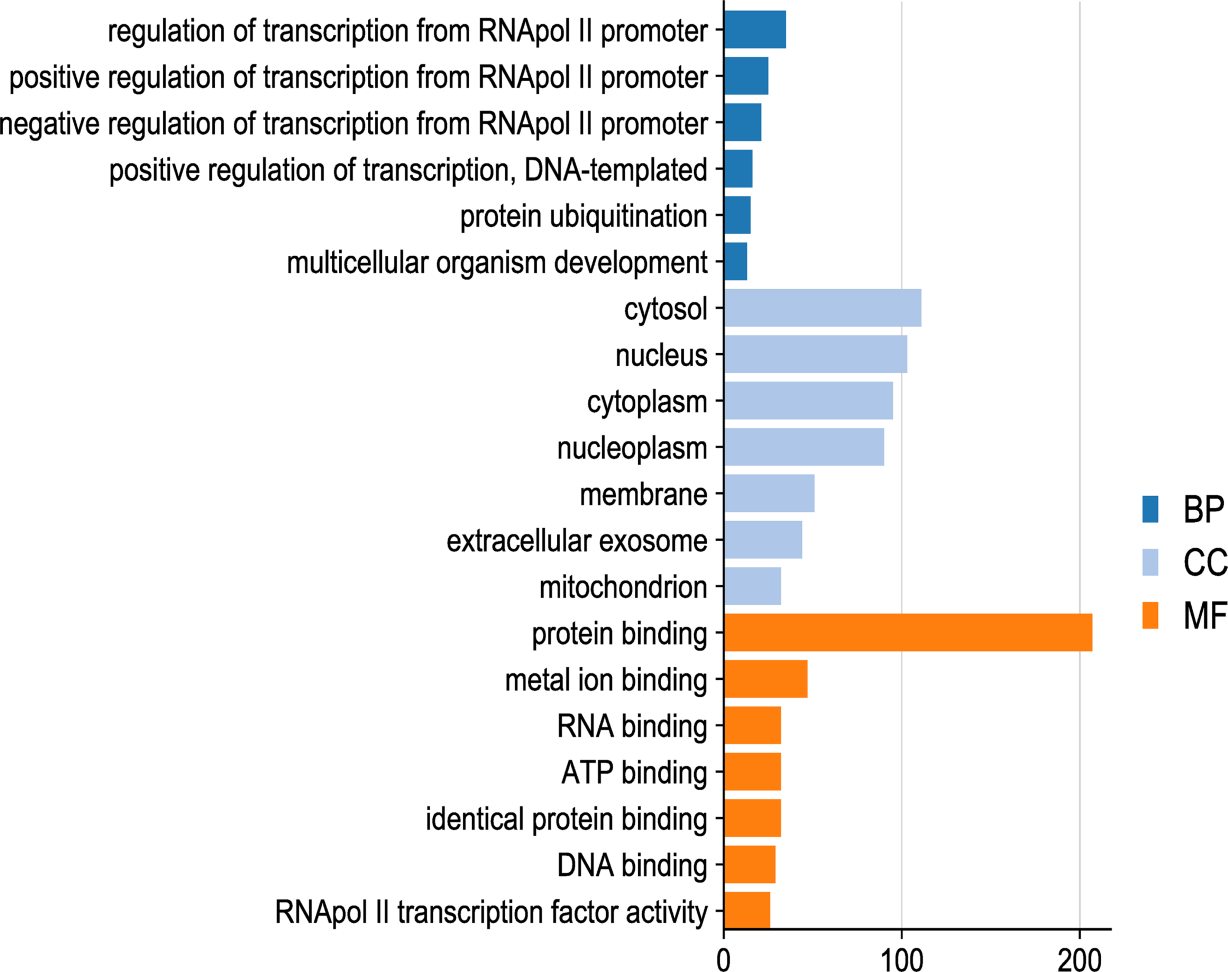
Gene Ontology (GO) analysis for all downregulated genes after EV-A71 infection on glioma cells. The distribution of downregulated genes from EV-A71 groups was obtained relative to the mock infection. GO analysis for all downregulated genes was performed and many enriched GO categories were found.

A total number of 441 genes was screened as upregulation (GFOLD value ≥ 1) and 320 genes as downregulation (GFOLD value ≤ −1) (**Tables S1**, **S2**). Since oncolytic viruses have a natural propensity to infect and kill tumor cells, the downregulated genes’ function after infection may contribute to enhance the malignant phenotype of gliomas. We performed Gene Ontology (GO) analysis for all downregulated genes and found many enriched GO categories (**Figure 1**). Furthermore, we found that ABCD3 was involved in several top and significantly enriched GO categories: cytosol, membrane, mitochondrion, protein binding, and ATP binding. Also, ABCD3 was among the top 5 downregulated genes in glioma cells after oncolytic virus EV-A71 infection (**Table S1**). However, the expression and correlation of ABCD3 with glioma have not been reported before.

### The mRNA and protein expression levels of ABCD3 are upregulated in glioma samples

**Figure 2:**
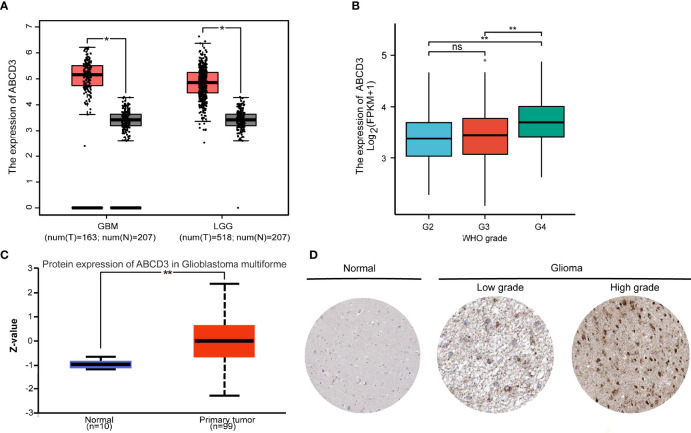
The mRNA and protein expression levels of ABCD3 are upregulated in glioma samples. **(A)** Differential expression of ABCD3 in both GBM and LGG compared with matched control samples. **(B)** Differential expression of ABCD3 in different glioma grades. **(C)** Differential ABCD3 protein expression levels in glioblastoma compared with normal tissues based on the data from the CPTAC. **(D)** Immunohistochemistry images of ABCD3 protein expression in glioma tissues and their normal controls. ns, no significance. *, *P* < 0.05; **, *P* < 0.01.

To examine the expression of ABCD3 in glioma progression, the data from publicly available datasets—TCGA, CGGA, GTEx, CPTAC, and HPA—were used to investigate the ABCD3 mRNA or protein expression patterns in glioma samples or normal tissue samples. Firstly, we compared the mRNA expression of ABCD3 in clinical brain tumors with different WHO-classified degrees in the TCGA database. In the TCGA database, brain tumors are divided into two cohorts: GBM (grade IV) and LGG (grades II and III). In general, ABCD3 was upregulated in both GBM and LGG compared with matched control samples (TCGA normal and GTEx data) (*P* < 0.01, **Figure 2A**). Analysis of the data from the CGGA showed similar results (**Supplementary Figure 2A**). In comparison among gliomas with WHO grades, ABCD3 was higher in grade IV than in grades II and III, and there was no statistical difference between grades II and III (**Figure 2B**).

We further checked ABCD3 protein expression levels. The protein expression level was also upregulated in glioblastoma multiforme in comparison with normal tissues based on the data from the CPTAC (**Figure 2C**), indicating that ABCD3 protein and mRNA expression levels were similar in different databases. Immunohistochemistry showed positive reactions of ABCD3 protein in cytoplasmic and membranous staining of both high- and low-grade gliomas. Meanwhile, ABCD3 displayed a strong intensity and over 75% quantity in high-grade glioma compared with a moderate intensity and around 50% quantity in low-grade glioma. In normal cerebral cortex tissue, ABCD3 has not been detected in the neuropil, endothelial cells, and neuronal cells and only showed low staining and less than 25% quantity in glial cells (**Figure 2D**).

### ABCD3 expression is associated with clinical characteristics of glioma patients

**Figure 3:**
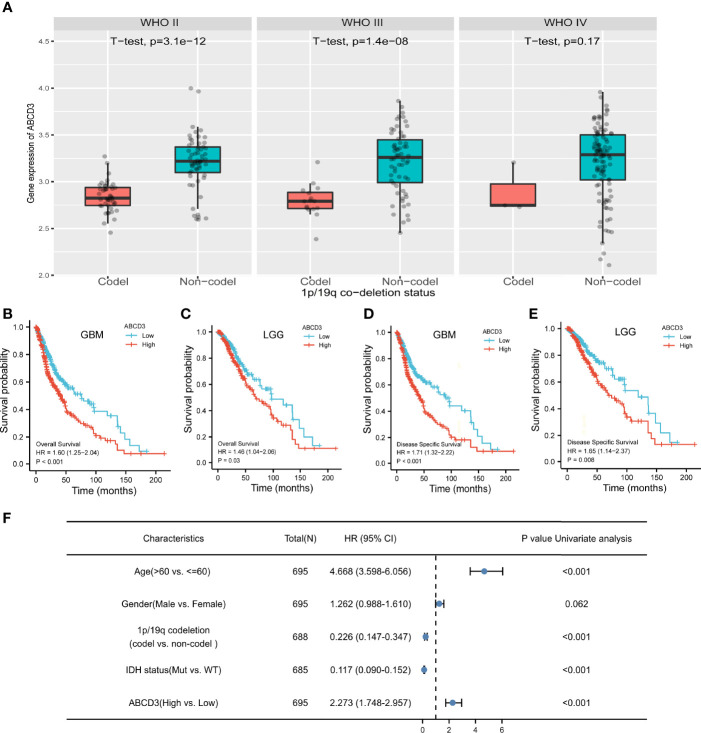
ABCD3 expression is associated with the clinical characteristics of glioma patients. **(A)** ABCD3 expression is lower in the 1p/19q co-deletion (Codel) groups than in the no co-deletion groups in all three WHO grade gliomas. **(B–E)** Overall survival and disease-specific survival curve of GBM and LGG patients based on differential ABCD3 expression. **(F)** Univariate Cox analysis of ABCD3 expression and other clinical pathological factors for GBM and LGG.

Glioma harboring the co-deletion status of 1p and 19q chromosome arms (1p/19q) shows favorable prognostic and predictive values. In the subgroup analysis, we noticed that ABCD3 expression is significantly lower in the co-deletion (Codel) group than in the no co-deletion groups in the WHO II and III grades (**Figure 3A**). However, ABCD3 did not display a statistically different expression in other glioma subgroups in terms of gender and age (**Supplementary Figure 3**).

For survival analysis, both GBM and LGG patients were divided separately into high and low ABCD3 groups based on the mean value of TPM. The cutoff value of the GBM group is 5.10 and that of the LGG group is 5.01. The survival curve showed that high ABCD3 expression significantly decreased the OS and DSS. For GBM, the median survival time of the low ABCD3 expression group was 76.1 versus 40.3 months of the high ABCD3 expression group. Meanwhile, for LGG, the median survival time of the low ABCD3 expression group was 135.6 versus 68.4 months of the high ABCD3 expression group. Patients with high ABCD3 expression had a worse prognosis.

The hazard ratio (HR) for the high ABCD3 expression on OS of GBM patients was 1.60 (95% CI, 1.25 to 2.04; *P* < 0.001) and that of LGG patients was 1.46 (95% CI, 1.04 to 2.06; *P* = 0.03). Additionally, the HR for high ABCD3 expression on DSS of GBM patients was 1.71 (95% CI, 1.32 to 2.22; *P* < 0.001), and that of LGG patients was 1.65 (95% CI, 1.14 to 2.37; *P* = 0.008) (**Figures 3B–E**). Analysis of the data from the CGGA showed similar results (**Supplementary Figure 2B**). Moreover, Cox regression analysis also revealed that age and IDH status as well as ABCD3 and 1p/19q are significantly associated with overall survival (**Figure 3F**).

### Relationship between ABCD3 expression and tumor-infiltrating immune cells

**Figure 4:**
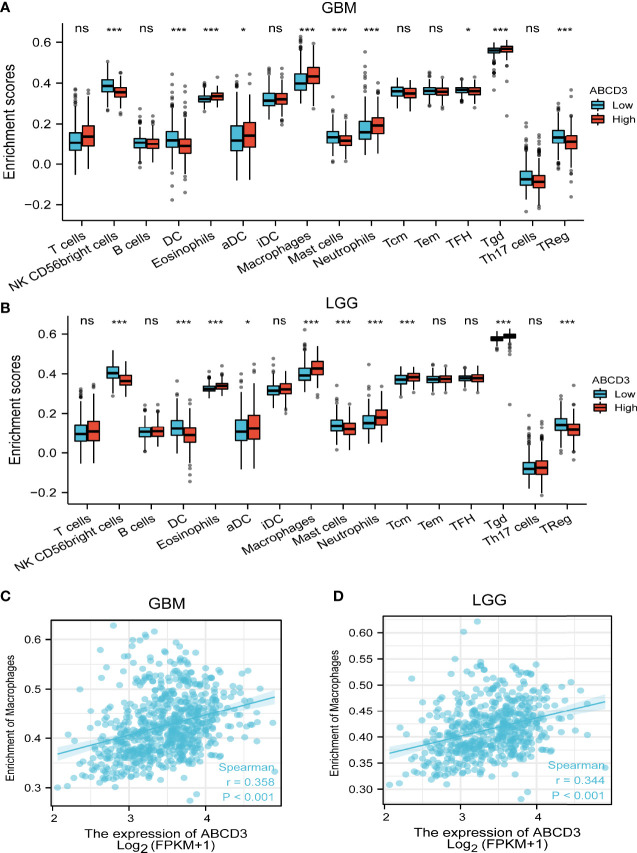
Relationship between ABCD3 expression and tumor-infiltrating immune cells. The proportion of 24 subpopulations of immune cell infiltration between the high and low expression of ABCD3 in GBM **(A)** or LGG **(B)** samples. Macrophages were mostly increased in the high ABCD3 expression group compared with the low ABCD3 expression group, while pDC cells were apparently decreased in the high expression group in both GBM and LGG. The macrophage infiltrate correlated with both GBM **(C)** and LGG **(D)**. ns, no significance; *, *P* < 0.05; ***, *P* < 0.001.

As tumor-infiltrating immune cells (IC) are associated with glioma prognosis (Sokratous et al., 2017), we conducted an analysis to find out if ABCD3 expression was associated with immune infiltration in GBM and LGG. All GBM and LGG samples were separately divided into high and low groups based on the mean value of ABCD3. We used an established computational resource ssGSEA to estimate the differing concentrations of 24 immune cell types in the high and low ABCD3 expression groups with CIBERSORT. The proportion of 24 subpopulations of immune cells appears clearly in **Figure 4**. The Wilcoxon signed-rank test was used to show the significant difference between the two groups. For GBM, macrophages, neutrophils, and Th2 cells were apparently increased in the high ABCD3 expression group. In contrast, DC, pDC, Th17 cells, and Treg cells were decreased in the high ABCD3 expression group (**Figure 4A**). The immune infiltration status of LGG displayed a similar pattern along with ABCD3 expression compared to GBM. However, the Th17 proportion in LGG was not associated with ABCD3 expression (**Figure 4B**). For macrophages that were significantly correlated with ABCD3, we provided a way to further display them in a scatter plot. The macrophage infiltrate correlated with both GBM and LGG, and correlations and *P*-values are included in **Figures 4C, D**.

### A correlation exists between ABCD3 expression and immune infiltration levels in glioma obtained from TIMER

**Figure 5:**
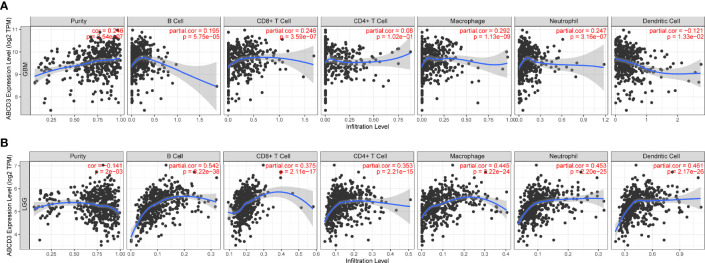
The ABCD3 expression level has a correlation with the tumor-infiltrating levels from TIMER. **(A)** ABCD3 expression has positive correlations with infiltrating levels of B cell, CD8^+^ T cell, macrophage, and neutrophil, while it has a negative correlation with dendritic cells in GBM. **(B)** ABCD3 expression has a significant positive correlation with all immune infiltration cells in LGG.

To ensure the accuracy of infiltration levels associated with ABCD3, we also assessed how ABCD3 expression correlates with immune infiltration levels in different grades of glioma using TIMER. The results showed a positive correlation between the high expression of ABCD3 and the high levels of all immune infiltration in LGG (**Figure 5B**). Compared with LGG, there was a weak significant correlation between immune infiltration and ABCD3 expression from TIMER. Moreover, a negative correlation exists between ABCD3 expression level and infiltrating levels of dendritic cells (*r* = −0.121, *P* = 1.33e−02) in GBM (**Figure 5A**).

### Gene sets enriched in the ABCD3 expression phenotype

In order to functionally annotate the potential functions or signaling pathways related to ABCD3 expression, pathway enrichment was assessed by GSEA. GBM and LGG samples were sorted into high and low expression groups, respectively, according to ABCD3 expression. The different transcriptional profiles were identified and sorted by log2FC with DESeq2. Then, we ran GSEA on these transcriptional profiles and assessed the enrichment using the NES as a metric of enrichment. The top 5 ABCD3-related items are listed and visualized in each group (**Table 1**).

**Table 1:** Gene sets enriched in different ABCD3 expression phenotypes.

Gene set name	NES	*P*-values	*q*-values
LGG high ABCD3 expression			
HALLMARK_G2M_CHECKPOINT	2.67	0.0034	0.0066
HALLMARK_INTERFERON_GAMMA_RESPONSE	2.16	0.0034	0.0066
HALLMARK_EPITHELIAL_MESENCHYMAL_TRANSITION	2.11	0.0034	0.0066
HALLMARK_TNFA_SIGNALING_VIA_NFKB	2.01	0.0034	0.0066
HALLMARK_INTERFERON_ALPHA_RESPONSE	2.00	0.0027	0.0066
LGG low ABCD3 expression			
HALLMARK_KRAS_SIGNALING_DN	−1.71	0.0014	0.0066
HALLMARK_MYOGENESIS	−1.50	0.0084	0.0119
HALLMARK_OXIDATIVE_PHOSPHORYLATION	−1.40	0.0167	0.0207
HALLMARK_SPERMATOGENESIS	−1.24	0.0897	0.0983
HALLMARK_CHOLESTEROL_HOMEOSTASIS	−1.24	0.0250	0.1268
GBM high ABCD3 expression			
WP_TYROBP_CAUSAL_NETWORK	2.73	0.0027	0.0197
PID_AURORA_B_PATHWAY	2.44	0.0025	0.0197
PID_PLK1_PATHWAY	2.43	0.0026	0.0197
WP_DNA_IRDAMAGE_AND_CELLULAR_RESPONSE_VIA_ATR	2.39	0.0027	0.0197
REACTOME_INTERLEUKIN_10_SIGNALING	2.38	0.0026	0.0197
GBM low ABCD3 expression			
REACTOME_NEURONAL_SYSTEM	−2.62	0.0013	0.0197
REACTOME_TRANSMISSION_ACROSS_CHEMICAL_SYNAPSES	−2.49	0.0013	0.0197
WP_SYNAPTIC_VESICLE_PATHWAY	−2.45	0.0015	0.0197
REACTOME_POTASSIUM_CHANNELS	−2.41	0.0015	0.0197
REACTOME_NEUROTRANSMITTER_RELEASE_CYCLE	−2.38	0.0015	0.0197

**Figure 6:**
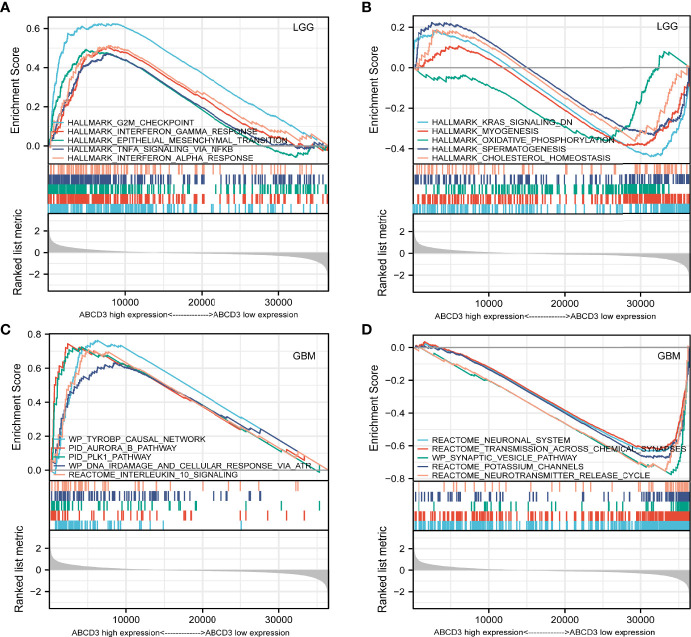
GSEA analysis of LGG or GBM samples with high or low ABCD3 expression phenotype. **(A)** GSEA results showing the top 5 enriched gene sets in the LGG samples with high ABCD3 expression based on NES. **(B)** GSEA results showing the top 5 enriched gene sets in the LGG samples with low ABCD3 expression based on NES. **(C)** GSEA results showing the top 5 enriched gene sets in the GBM samples with high ABCD3 expression based on NES. **(D)** GSEA results showing the top 5 enriched gene sets in the GBM samples with low ABCD3 expression based on NES.

For the LGG samples with high ABCD3 expression, GSEA confirmed the strong enrichment for inflammatory genes among the differentially expressed pathways including interferon-gamma response, epithelial–mesenchymal transition (EMT), TNF-α signaling, and interferon-α response (**Table 1** and **Figure 6A**). The inflammatory response and EMT are the hallmarks of many cancer types. K-ras signaling, oxidative phosphorylation, and cholesterol homeostasis gene sets were significantly enriched in the low ABCD3 expression phenotype for LGG (**Figure 6B**). These indicated the potential role of ABCD3 in the development of LGG.

Gene sets including the plk1 pathway, tyrobp causal network, ir-damage and cellular response, and interleukin-10 signaling showed significant differential enrichment in the high ABCD3 expression GBM phenotype (**Figure 6C**). Meanwhile, the neuronal system, transmission across chemical synapses, synaptic vesicle pathway, and potassium channels showed significant differential enrichment in the low ABCD3 expression GBM group based on NES (**Table 1** and **Figure 6D**).

## Discussion

ABCD3 is involved in the transport of fatty acids in the peroxisome and may play an important role in tumorigenesis (Reams et al., 2015). ABCD3 has been shown to be a biogenetic marker for malignancies such as human prostate cancer (Zhang et al., 2020b), ovarian cancer (Lima et al., 2015; Elsnerova et al., 2016), and non-small cell lung cancer (Tran, 2013) but has hardly been evaluated in glioma. Here, we screened ABCD3 as one of the most dramatically downregulated genes during oncolytic virus EV-A71 treatment. It is believed that oncolytic viruses mediate antitumor effects mainly through the following mechanisms: 1) specifically replicate in tumor cells with direct lysis and 2) release viral particles from lysed tumor cells to stimulate systemic immunity, such as promoting tumor antigen presentation, increasing the tumor microenvironment immune cell infiltration, modulating the tumor microenvironment, activating the immune cells, and activating the body’s immune system through the immunomodulatory factors carried by them. In addition, it has been shown that some viruses can indirectly inhibit the immune system by infecting tumor-related vascular endothelial cells and also inhibit tumor angiogenesis. Therefore, during the lysis of gliomas by oncolytic viruses, significantly downregulated genes during the lysis of gliomas induced by oncolytic viruses are often necessary for the malignant proliferation of gliomas.

In the GO analysis for all downregulated genes, we also found that ABCD3 was involved in several top and significantly enriched GO categories. Oncolytic viruses cause glioma cell death through different mechanisms, including pyroptosis, necroptosis, and apoptosis. Genes that were downregulated in this process are often associated with glioma proliferation and malignancy. Oncolytic viruses also trigger an inflammatory immune response in the tumor microenvironment (TME). Gliomas that metastasize to the central nervous system (CNS) are characterized by the highly immunosuppressive TME. Therefore, ABCD3 is likely to be a diagnostic and prognostic biomarker associated with the clinical features and immune infiltration of gliomas.

Consequently, we conducted the first-ever analysis of ABCD3 expression in large cohorts of human glioma patients, which unveiled the potential role of ABCD3 in glioma. Based on RNA-seq analysis, protein analysis, and clinical data, we performed a retrospective analysis on glioma patients with histological confirmation. We found that ABCD3 expression is associated with tumor grade in glioma patients as ABCD3 is higher in grade IV than in grades II and III. In gliomas, the co-deletion status of 1p and 19q chromosome arms is associated with the oligodendrocyte component of the tumor, and co-deletion abnormalities of both are detectable in 70% of low-grade gliomas and 60% of mesenchymal oligodendrogliomas. The 1p/19q co-deletion patients respond well to radiotherapy and chemotherapy, suggesting a longer survival and a relatively good prognosis. In the subgroup analysis, we noticed that ABCD3 expression was significantly lower in the co-deletion group than in the no co-deletion groups in all three WHO grades. Similarly, we found that ABCD3 expression levels in gliomas were related to prognosis. In gliomas, high ABCD3 expression was also associated with a poor prognosis. ABCD3 expression was found to be an independent prognostic factor of glioma patient prognosis in multivariate analyses. Cox regression analysis also revealed that age and IDH status as well as ABCD3 were significantly associated with overall survival.

Multiple diseases of the CNS, including malignant diseases, are associated with dysregulation of inflammatory responses (Pedemonte et al., 2006). Since gliomas are largely protected from immune cells infiltrating them, they have long been considered immune-inaccessible to antitumor-immune (Roesch et al., 2018). Given that oncolytic virus treatment could trigger an inflammatory immune response in the glioma microenvironment (Walton et al., 2018), a systematic analysis of the correlation between ABCD3 and tumor immunity was performed. Our studies showed that ABCD3 expression could affect the immune infiltration levels and diverse immune marker sets in glioma. A positive correlation was found between ABCD3 and macrophages and active dendritic cells in the microenvironment of both GBM and LGG. They provided a supportive stroma for the proliferation and invasion of neoplastic cells. These results may explain why high ABCD3 gliomas progressed malignantly and had adverse outcomes. ABCD3 was also closely related to glioma purity and immune score in LGG.

A lower purity indicates a poor prognosis and enhances the malignancy progression phenotype. High ABCD3 gliomas are more likely to occur in tissues with more complex microenvironments, based on this result.

GSEA also confirmed the strong enrichment for inflammatory genes among the differentially expressed pathways including the EMT, interferon-gamma response, TNF-α signaling, and interferon-α response. The inflammatory response and EMT are the hallmarks of many cancer types (Suarez-Carmona et al., 2017; Cho et al., 2019). K-ras signaling, oxidative phosphorylation, and cholesterol homeostasis gene sets were significantly enriched in the low ABCD3 expression phenotype for LGG. These indicated the potential role of ABCD3 in the development of LGG.

In conclusion, we screened an unreported ABCD3 gene by high-throughput bioinformatics analysis of genes significantly altered during treatment of gliomas with the oncolytic viruses EV-A71. Using multiple databases, we comprehensively analyzed the correlation between ABCD3 mRNA and protein expression levels and clinical glioma patient characteristics, immune infiltration, gene enrichment analysis, etc. We conclude that ABCD3 is highly expressed in different degrees of gliomas and is associated with poor prognosis and many clinical characteristics. We also found that ABCD3 expression could affect the immune infiltration levels and diverse immune marker sets in glioma and, especially, could enhance the malignancy progression phenotype of LGG. Our results suggested that ABCD3 could be a potential biomarker for glioma prognosis and immunotherapy response. Our results also further enriched the theoretical and molecular mechanisms of oncolytic virus treatment for malignant gliomas.

## Data Availability Statement

Publicly available datasets were analyzed in this study. The names of the repository/repositories and accession number(s) can be found in the article/**Supplementary Material**

## Author Contributions

Conceptualization: JL and XY. Resources: XY. Data curation: JL, YZ, and XY. Software: ZQ. Formal analysis: JL, YZ, RD, and XY. Supervision: RD. Funding acquisition: XY. Validation: XY. Investigation: JL and YZ. Visualization: JL and XY. Methodology: RD and XY. Writing—original draft: JL. Project administration: XY. Writing—review and editing: XY. All authors read and approved the final manuscript. All authors contributed to the article and approved the submitted version.

## Funding

This research was funded by the Applied Basic Research Project of Shanxi Province (20210302123266).

## Conflict of Interest

The authors declare that the research was conducted in the absence of any commercial or financial relationships that could be construed as a potential conflict of interest.

## Publisher’s Note

All claims expressed in this article are solely those of the authors and do not necessarily represent those of their affiliated organizations, or those of the publisher, the editors and the reviewers. Any product that may be evaluated in this article, or claim that may be made by its manufacturer, is not guaranteed or endorsed by the publisher.

## Supplementary Material

Supplementary Figure 1 A high level of quality and sufficient quantity of data was tested by FastQC. The FastQC results confirmed a high level of quality and sufficient quantity for further gene functional analysis.

Supplementary Figure 2 ABCD3 expression is associated with glioma grades and survival rate from CGGA. **(A)** ABCD3 expression is associated with glioma grades. **(B)** Overall survival curve of all grade primary glioma based on differential ABCD3 expression.

Supplementary Figure 3 ABCD3 different expression with glioma subgroups. ABCD3 did not display a statistical different expression in patient Gender **(A)** and Age **(B)**.
