# Supplementary figures and images for: ABCD3 is a prognostic biomarker for glioma and associated with immune infiltration: A study based on oncolysis of gliomas
Source: Front Cell Infect Microbiol. 2022 Jul 25;12:956801. doi: 10.3389/fcimb.2022.956801 (PMC9358688; doi:10.3389/fcimb.2022.956801)

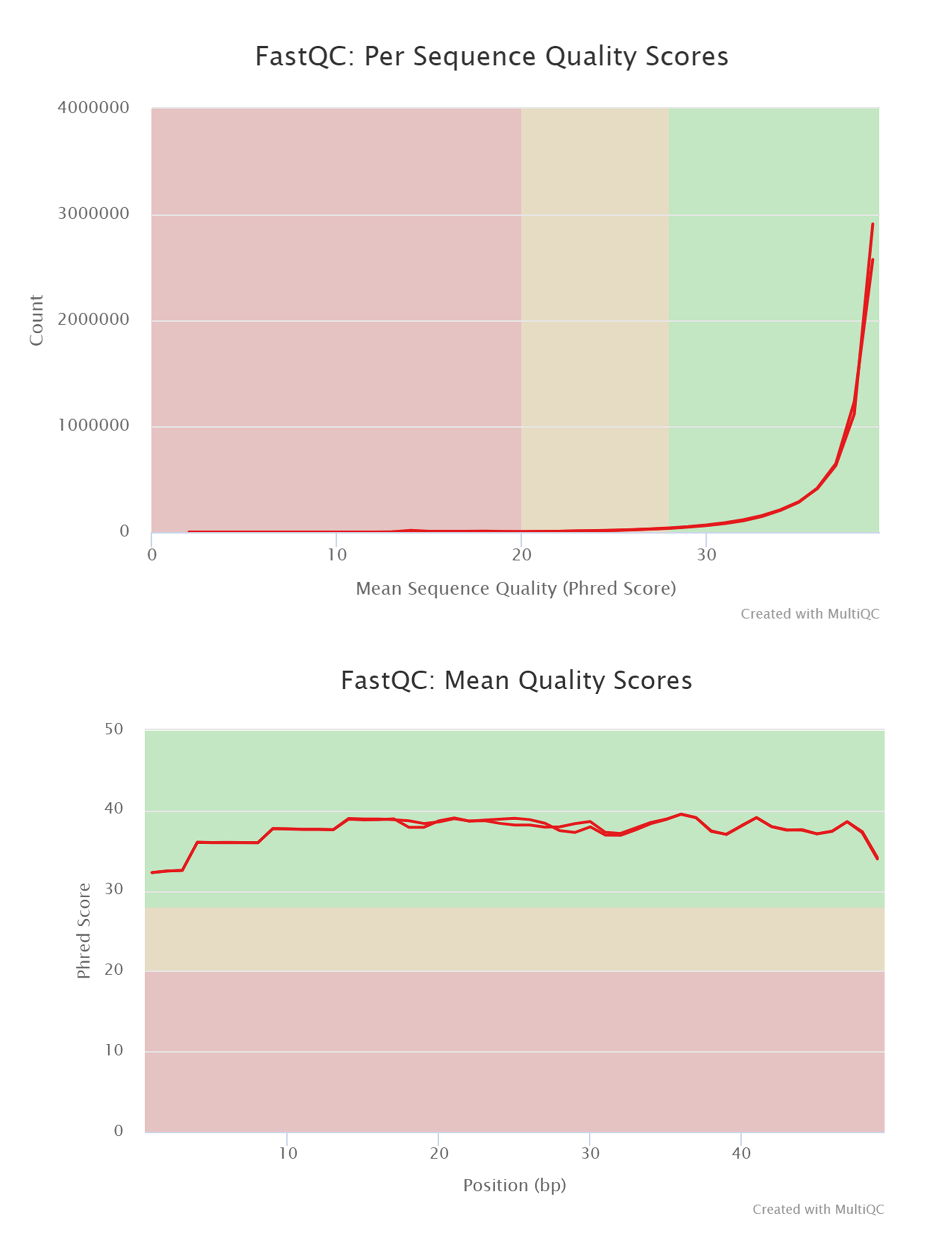

Supplement: Supplementary Figure 1 — A high level of quality and sufficient quantity of data was tested by FastQC. The FastQC results confirmed a high level of quality and sufficient quantity for further gene functional analysis. [file Image_1.tif]

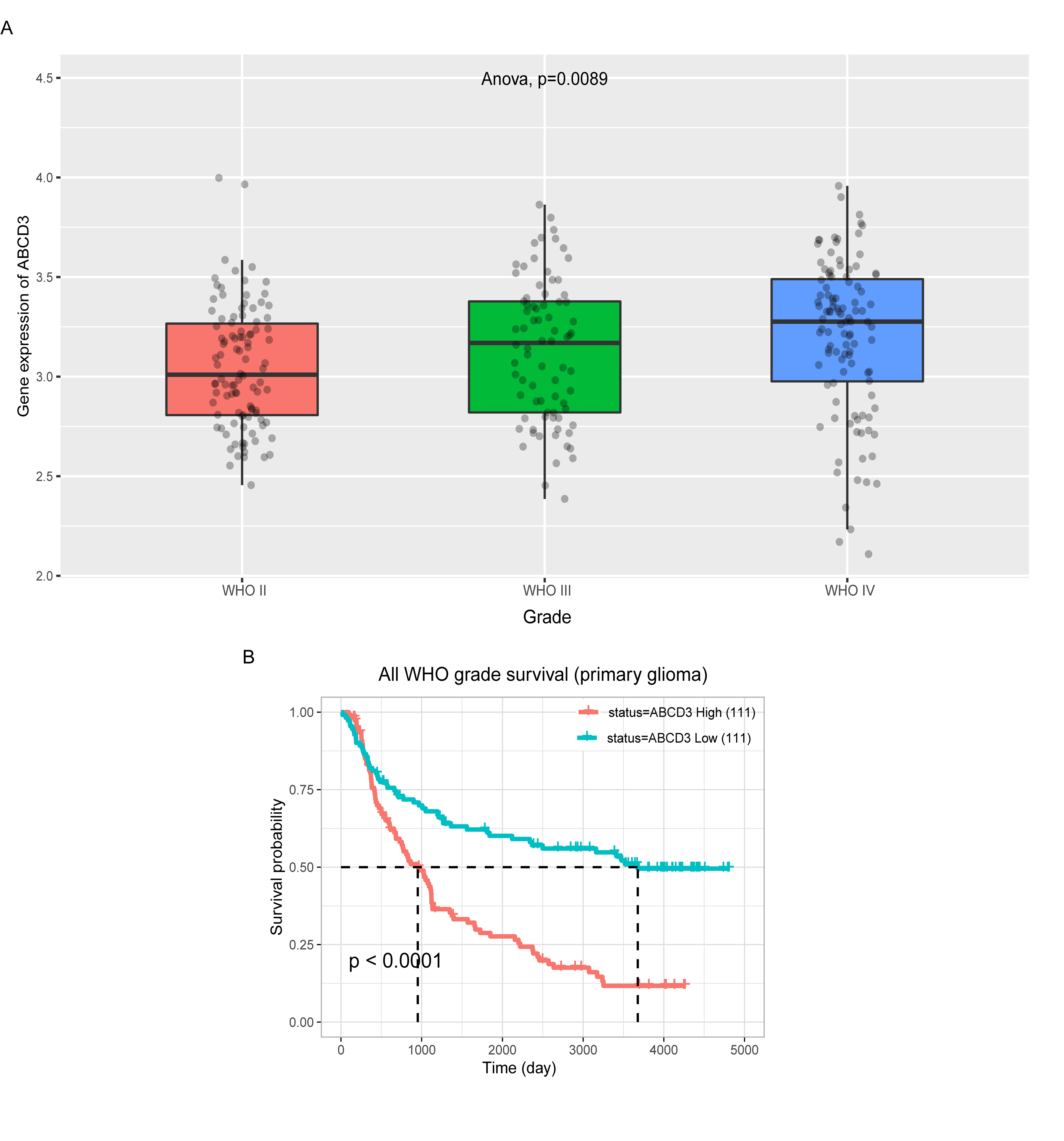

Supplement: Supplementary Figure 2 — ABCD3 expression is associated with glioma grades and survival rate from CGGA. (A) ABCD3 expression is associated with glioma grades. (B) Overall survival curve of all grade primary glioma based on differential ABCD3 expression. [file Image_2.tif]

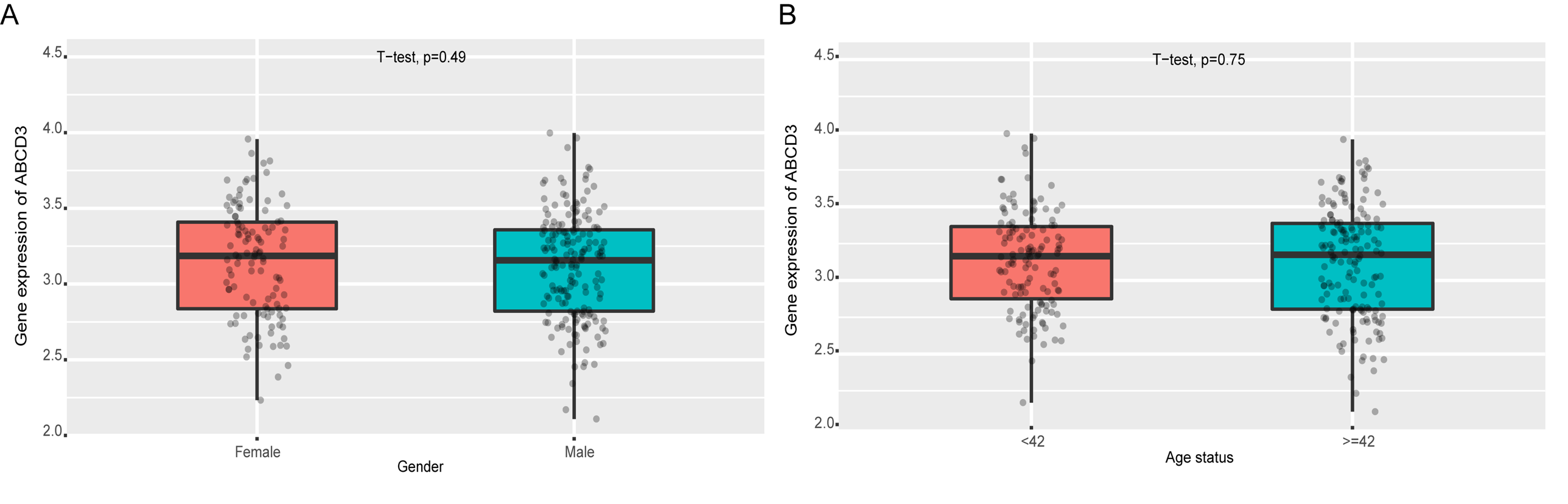

Supplement: Supplementary Figure 3 — ABCD3 different expression with glioma subgroups. ABCD3 did not display a statistical different expression in patient Gender (A) and Age (B). [file Image_3.tif]
