# Supplementary material for: ABCD3 is a prognostic biomarker for glioma and associated with immune infiltration: A study based on oncolysis of gliomas
Source: Front Cell Infect Microbiol. 2022 Jul 25;12:956801. doi: 10.3389/fcimb.2022.956801 (PMC9358688; doi:10.3389/fcimb.2022.956801)
Supplement: Supplementary file 4 [file Table_1.docx]

**Table S1 Down-regulated genes after EV-A71 infection**

| **GeneSymbol** | **GFOLD** | **log2fdc** |
| --- | --- | --- |
| HSPA2 | -2.8202 | -3.09248 |
| PPP1R3C | -2.71197 | -3.38873 |
| HS6ST1 | -2.70418 | -2.94216 |
| PGRMC1 | -2.60448 | -2.99154 |
| ABCD3 | -2.46471 | -2.94844 |
| NDUFS7 | -2.45165 | -2.83629 |
| RALGDS | -2.4268 | -3.25293 |
| AP1S1 | -2.36281 | -2.74203 |
| WDR67 | -2.31209 | -4.00629 |
| THAP7 | -2.23459 | -2.70507 |
| GDF5 | -2.19526 | -2.79607 |
| TIGD5 | -2.19121 | -2.99401 |
| CALM2 | -2.10741 | -2.23901 |
| ARHGAP33 | -2.10522 | -2.70861 |
| SIGMAR1 | -2.10375 | -2.42727 |
| CDC42EP1 | -2.07845 | -2.33539 |
| MRPL16 | -2.06935 | -2.53528 |
| UFC1 | -2.05988 | -2.26026 |
| ALKBH7 | -2.05711 | -2.45626 |
| ZBTB4 | -2.03706 | -2.47768 |
| RPS20 | -2.0309 | -2.11737 |
| MVP | -2.01132 | -2.22554 |
| APOBEC3B | -1.99283 | -2.30101 |
| CASD1 | -1.97583 | -2.52138 |
| PLA2G16 | -1.94958 | -2.54883 |
| CNO | -1.94251 | -2.67208 |
| ZYG11B | -1.92849 | -2.28596 |
| PTBP1 | -1.91135 | -2.09173 |
| DGKD | -1.88926 | -2.50884 |
| C13orf15 | -1.87778 | -2.07517 |
| NLRX1 | -1.85235 | -2.51596 |
| MLLT11 | -1.8452 | -2.4479 |
| MRPL49 | -1.82532 | -2.35668 |
| PI15 | -1.80828 | -2.06037 |
| SCAND2 | -1.80482 | -2.83789 |
| ZWINT | -1.77638 | -2.01986 |
| RPL22 | -1.77134 | -1.91838 |
| PSMG3 | -1.77025 | -2.08365 |
| PAK4 | -1.76042 | -2.40493 |
| ZNF322A | -1.75344 | -3.10093 |
| PARG | -1.74595 | -2.24259 |
| LIMCH1 | -1.74338 | -2.03637 |
| TOP2A | -1.74022 | -1.88562 |
| ZNF323 | -1.74005 | -2.32955 |
| XPO6 | -1.72515 | -2.03269 |
| GPX1 | -1.72159 | -1.87176 |
| CSTF1 | -1.71828 | -2.30063 |
| BIVM | -1.71341 | -2.5699 |
| PIF1 | -1.70252 | -2.44196 |
| SNRPD3 | -1.68714 | -2.02279 |
| POLR3GL | -1.67903 | -2.13603 |
| LOXL1 | -1.67846 | -1.80986 |
| IER5L | -1.67777 | -2.50302 |
| IL10RB | -1.6721 | -2.08558 |
| HEXIM1 | -1.66834 | -2.37846 |
| EZH1 | -1.6553 | -2.4335 |
| C17orf79 | -1.65444 | -2.02733 |
| P2RX6 | -1.65254 | -2.26221 |
| SAMM50 | -1.64297 | -2.09743 |
| INTS9 | -1.62776 | -2.89448 |
| C7orf47 | -1.6164 | -1.94026 |
| CDCA7 | -1.60887 | -2.10513 |
| UQCRQ | -1.60682 | -1.7294 |
| RANBP9 | -1.60415 | -1.9619 |
| RPL38 | -1.60256 | -1.70036 |
| CPSF3 | -1.59905 | -1.92584 |
| FAM160B2 | -1.5975 | -2.03967 |
| FOXG1 | -1.59286 | -1.89674 |
| TSHZ1 | -1.58771 | -3.3331 |
| AZI1 | -1.58464 | -2.17991 |
| IDH1 | -1.58347 | -1.99154 |
| PDXP | -1.57756 | -2.14301 |
| PGLS | -1.56671 | -1.84194 |
| TWSG1 | -1.5627 | -1.87144 |
| C7orf11 | -1.56091 | -1.97748 |
| MTA3 | -1.55489 | -2.31866 |
| PDE5A | -1.55376 | -1.649 |
| LOC253039 | -1.55173 | -3.03054 |
| GCLC | -1.54787 | -1.86596 |
| MOBKL2A | -1.54303 | -2.13995 |
| RNF40 | -1.54085 | -1.98803 |
| HIRA | -1.5381 | -2.2145 |
| HRAS | -1.5364 | -1.80148 |
| HMGN1 | -1.52661 | -1.68275 |
| AKT1S1 | -1.52327 | -1.98505 |
| RNF20 | -1.51143 | -2.118 |
| ZNF32 | -1.51077 | -1.99685 |
| RPL41 | -1.50425 | -1.57279 |
| EIF4E2 | -1.49898 | -2.06059 |
| ORC3 | -1.49547 | -2.24466 |
| ARFRP1 | -1.49302 | -1.97971 |
| NENF | -1.49141 | -1.75211 |
| MOGS | -1.48152 | -1.90316 |
| PYGB | -1.47957 | -1.857 |
| SRSF6 | -1.46967 | -1.85764 |
| CDK1 | -1.46901 | -1.64113 |
| ORAI3 | -1.46696 | -2.12365 |
| RXRA | -1.4572 | -1.80724 |
| RNF34 | -1.45033 | -1.9474 |
| FAIM | -1.44461 | -1.93298 |
| TPRN | -1.44421 | -2.01486 |
| C9orf150 | -1.44172 | -1.87614 |
| ZNF580 | -1.43097 | -1.86879 |
| HDGF | -1.43022 | -1.59031 |
| KIAA0528 | -1.42783 | -2.00629 |
| IRF2BP1 | -1.42746 | -1.8326 |
| CENPB | -1.42294 | -1.66564 |
| TRAM2 | -1.42221 | -1.65361 |
| CCDC97 | -1.41978 | -1.90904 |
| ARL8A | -1.41966 | -1.7839 |
| TRIP6 | -1.41097 | -1.72761 |
| MYL6 | -1.41062 | -1.48114 |
| THBD | -1.40599 | -3.16804 |
| PBX1 | -1.4055 | -1.94565 |
| FBXL15 | -1.40467 | -2.83199 |
| DNAJC17 | -1.40358 | -2.72851 |
| HSD17B10 | -1.40236 | -1.79014 |
| DEK | -1.39543 | -1.53816 |
| HIF1A | -1.39132 | -1.48391 |
| HOXA3 | -1.38622 | -2.87853 |
| BCL7C | -1.38592 | -1.86389 |
| SARS2 | -1.38501 | -2.12365 |
| DYNC2LI1 | -1.37307 | -2.94127 |
| SEZ6L2 | -1.37135 | -2.11071 |
| YEATS4 | -1.36831 | -1.9421 |
| CHRAC1 | -1.36135 | -1.69925 |
| PNPLA6 | -1.35787 | -1.5998 |
| MYST4 | -1.35343 | -2.27629 |
| DNAJA3 | -1.35093 | -1.70861 |
| PON2 | -1.34827 | -1.56105 |
| DSCR6 | -1.34625 | -1.7161 |
| LCMT2 | -1.34611 | -2.3301 |
| PARL | -1.34371 | -1.70159 |
| STK32C | -1.34062 | -1.67239 |
| CDC23 | -1.3387 | -1.73945 |
| PPOX | -1.33659 | -1.98872 |
| PSRC1 | -1.33316 | -1.6613 |
| PRPF19 | -1.33308 | -1.65051 |
| IFT172 | -1.33266 | -2.99401 |
| YPEL3 | -1.32958 | -2.07118 |
| RAB5B | -1.32347 | -1.73681 |
| MPP1 | -1.32256 | -2.16804 |
| ARL5A | -1.32206 | -1.85977 |
| GGCT | -1.31993 | -1.555 |
| C1S | -1.31753 | -1.49432 |
| CHMP4A | -1.31623 | -1.62645 |
| HOXA11 | -1.31342 | -2.09004 |
| MEIR5 | -1.31058 | -2.478 |
| PSENEN | -1.3076 | -1.69612 |
| HCG11 | -1.30585 | -3.07784 |
| CIT | -1.30425 | -1.71323 |
| SSR2 | -1.3039 | -1.58014 |
| TOPBP1 | -1.30328 | -1.67439 |
| ATG4D | -1.30106 | -2.04421 |
| MEGF9 | -1.30034 | -1.96495 |
| DBI | -1.297 | -1.52437 |
| C4orf46 | -1.29651 | -1.68749 |
| NDUFS5 | -1.29364 | -1.46296 |
| MPG | -1.29273 | -1.67963 |
| C7orf26 | -1.29117 | -1.6155 |
| UBE2C | -1.29076 | -1.47995 |
| PGGT1B | -1.28814 | -1.88793 |
| HSPA8 | -1.2861 | -1.39464 |
| SEC23IP | -1.28587 | -1.67506 |
| ERF | -1.27841 | -1.90416 |
| C6orf47 | -1.27694 | -1.84106 |
| SCFD2 | -1.27558 | -2.09688 |
| LOC729678 | -1.27468 | -2.00406 |
| SGSH | -1.27306 | -1.80172 |
| IARS2 | -1.27231 | -1.60081 |
| KIAA2013 | -1.27063 | -1.62736 |
| SDCCAG8 | -1.26826 | -2.84611 |
| FAM78B | -1.26826 | -2.84611 |
| CLCA2 | -1.26821 | -1.75462 |
| BTBD6 | -1.2647 | -1.64448 |
| CBLN2 | -1.26287 | -2.3331 |
| ABCA2 | -1.26281 | -2.55661 |
| IVNS1ABP | -1.26096 | -1.45778 |
| NEURL1B | -1.25522 | -1.827 |
| ELP2 | -1.25166 | -1.70861 |
| AP2S1 | -1.25018 | -1.52618 |
| ZNF689 | -1.24849 | -2.91806 |
| WBSCR22 | -1.24742 | -1.43025 |
| PPP1R12B | -1.24561 | -1.70278 |
| C9orf16 | -1.24189 | -1.48034 |
| SLC5A5 | -1.24125 | -2.68261 |
| SP3 | -1.23999 | -1.56937 |
| ACVR1 | -1.23408 | -1.75393 |
| CDCA3 | -1.23349 | -1.50915 |
| TERT | -1.23229 | -2.73836 |
| HDAC4 | -1.23225 | -1.81204 |
| CNNM4 | -1.2291 | -2.03054 |
| CEP120 | -1.22889 | -1.76488 |
| MPRIP | -1.22768 | -1.42692 |
| DDR1 | -1.22631 | -1.74648 |
| ZNF768 | -1.22313 | -1.83414 |
| METTL11A | -1.2173 | -1.63165 |
| FOXF2 | -1.21705 | -1.48748 |
| EPHX1 | -1.21676 | -1.32811 |
| SCARB1 | -1.2059 | -1.76045 |
| PGAM1 | -1.19779 | -1.26183 |
| DYNC2H1 | -1.19575 | -2.13007 |
| CASKIN2 | -1.19452 | -1.99812 |
| SATB2 | -1.19208 | -1.47318 |
| USP5 | -1.18814 | -1.49351 |
| KAZN | -1.18243 | -2.62917 |
| METRN | -1.18057 | -1.37394 |
| C2 | -1.17824 | -1.52696 |
| ATP5B | -1.17663 | -1.25963 |
| ACAD11 | -1.1758 | -1.70861 |
| PDGFRA | -1.1719 | -1.49828 |
| TMEM100 | -1.16678 | -1.61698 |
| GALNT7 | -1.16257 | -1.32301 |
| CDH11 | -1.16189 | -1.43998 |
| HCN2 | -1.16161 | -1.42005 |
| CCDC77 | -1.15855 | -1.77274 |
| BIN1 | -1.1574 | -1.41477 |
| UCK1 | -1.15612 | -1.66467 |
| SOX2 | -1.15467 | -1.478 |
| JUB | -1.15333 | -1.60085 |
| WNT5A | -1.14943 | -1.36515 |
| C16orf88 | -1.14728 | -1.73836 |
| CHSY1 | -1.14484 | -1.50062 |
| RPS13 | -1.14459 | -1.29904 |
| RPAIN | -1.14177 | -1.40365 |
| PPAPDC2 | -1.14142 | -2.931 |
| SEMA4B | -1.14106 | -1.94807 |
| ANKRD11 | -1.13862 | -1.8117 |
| INPPL1 | -1.13841 | -1.57736 |
| NAGLU | -1.13484 | -1.58308 |
| ZNF746 | -1.13196 | -1.47634 |
| MAP7D1 | -1.12928 | -1.32503 |
| TP53I11 | -1.12644 | -1.2474 |
| ISCU | -1.12508 | -1.48622 |
| MUM1 | -1.12351 | -1.60364 |
| UMPS | -1.11999 | -1.52339 |
| PERP | -1.1199 | -1.31812 |
| NDUFB2 | -1.11955 | -1.3 |
| FADD | -1.1186 | -1.68999 |
| MRPL40 | -1.11748 | -1.34438 |
| PTGFR | -1.11599 | -1.52895 |
| PSMB4 | -1.11584 | -1.31598 |
| PARP16 | -1.11566 | -2.70861 |
| TOM1 | -1.115 | -1.90362 |
| PLCD3 | -1.11427 | -1.64448 |
| IPO11 | -1.11427 | -1.64448 |
| METTL9 | -1.11052 | -1.2516 |
| SFT2D3 | -1.10642 | -2.18974 |
| RPS9 | -1.10456 | -1.24526 |
| CUEDC1 | -1.10409 | -1.8289 |
| CIRBP | -1.10045 | -1.34825 |
| SPNS1 | -1.09968 | -1.60604 |
| CSE1L | -1.09934 | -1.36661 |
| C5orf38 | -1.09878 | -1.67113 |
| ITPKB | -1.0986 | -1.7739 |
| NALCN | -1.09855 | -1.81015 |
| MAN1C1 | -1.09844 | -1.55788 |
| PSMB7 | -1.09816 | -1.2395 |
| SH3BP4 | -1.09556 | -1.37753 |
| MINK1 | -1.0947 | -1.47237 |
| FOXM1 | -1.09065 | -1.2505 |
| HRCT1 | -1.0895 | -2.54511 |
| NFE2L2 | -1.0872 | -1.22767 |
| CCT4 | -1.08477 | -1.31041 |
| LTB4R | -1.08263 | -2.16804 |
| RRAGC | -1.08187 | -1.70861 |
| CDCA2 | -1.08031 | -1.66035 |
| INO80E | -1.07953 | -1.61131 |
| HOXA10 | -1.0776 | -1.51306 |
| DMRTA2 | -1.07712 | -1.68724 |
| SC5DL | -1.07487 | -2.67208 |
| GYG2 | -1.07407 | -1.97164 |
| XIST | -1.07297 | -1.26443 |
| PIGC | -1.06925 | -1.59125 |
| MRPL24 | -1.06833 | -1.29704 |
| LGR4 | -1.06531 | -1.40358 |
| FIBIN | -1.06402 | -2.753 |
| ADAMTS1 | -1.06402 | -2.753 |
| C2orf7 | -1.06068 | -1.58308 |
| RAB40C | -1.05545 | -1.61091 |
| EIF4B | -1.05406 | -1.20465 |
| E2F2 | -1.05173 | -2.36396 |
| UBA7 | -1.05106 | -1.55506 |
| SLC10A3 | -1.05007 | -1.27956 |
| DCAF13 | -1.04755 | -1.68515 |
| CHST14 | -1.04405 | -1.46213 |
| SDSL | -1.04025 | -1.63672 |
| MFSD5 | -1.0384 | -1.62055 |
| RHOBTB3 | -1.03787 | -1.3388 |
| LRRFIP1 | -1.03565 | -1.44209 |
| SEPHS2 | -1.03462 | -1.38201 |
| SERTAD4 | -1.03293 | -2.63461 |
| TGIF2 | -1.03146 | -1.86907 |
| MAFG | -1.02778 | -1.46807 |
| CDH24 | -1.02721 | -1.60312 |
| CLSTN3 | -1.0269 | -1.52758 |
| ZDHHC23 | -1.02628 | -2.34088 |
| C9orf89 | -1.02599 | -1.57696 |
| EPHB4 | -1.02591 | -1.54991 |
| NAPRT1 | -1.02591 | -1.54991 |
| ATG16L2 | -1.02408 | -2.48622 |
| DVL1 | -1.02178 | -1.35753 |
| CLDN23 | -1.02008 | -1.64149 |
| CEBPG | -1.02007 | -1.46664 |
| TRIM2 | -1.01737 | -1.53684 |
| EDEM2 | -1.01664 | -1.55137 |
| C20orf43 | -1.0154 | -1.29357 |
| CLIC1 | -1.01482 | -1.14095 |
| TAF4 | -1.01463 | -1.3558 |
| U2AF1 | -1.01449 | -1.16303 |
| CEP170 | -1.01433 | -1.36238 |
| AGK | -1.01175 | -1.23196 |
| PPP4R1 | -1.0075 | -1.27449 |
| SRXN1 | -1.00685 | -1.2104 |
| 2-Sep | -1.0067 | -1.15911 |
| ZNF598 | -1.00331 | -1.23106 |
| BRD7P3 | -1.00247 | -1.50425 |
| AGR2 | -1.00124 | -1.2249 |
| GINS1 | -1.00123 | -1.40009 |
