# Supplementary material for: ABCD3 is a prognostic biomarker for glioma and associated with immune infiltration: A study based on oncolysis of gliomas
Source: Front Cell Infect Microbiol. 2022 Jul 25;12:956801. doi: 10.3389/fcimb.2022.956801 (PMC9358688; doi:10.3389/fcimb.2022.956801)
Supplement: Supplementary file 5 [file Table_2.docx]

**Table S2 Up-regulated genes after EV-A71 infection**

| **GeneSymbol** | **GFOLD** | **log2fdc** |
| --- | --- | --- |
| IL8 | 8.8565 | 9.5139 |
| CXCL3 | 8.67916 | 10.289 |
| IL6 | 7.3346 | 8.00804 |
| IFIT2 | 6.9771 | 7.31911 |
| IFIT3 | 5.85119 | 6.24559 |
| PTGS2 | 5.7612 | 6.01188 |
| RSAD2 | 5.55895 | 6.00159 |
| ATF3 | 5.3327 | 6.70361 |
| - | 5.27762 | 6.06398 |
| OASL | 5.12103 | 5.63653 |
| RND3 | 5.09407 | 5.79166 |
| TNFAIP3 | 4.89655 | 6.27248 |
| NFKB2 | 4.62518 | 5.11995 |
| IFIT1 | 4.61664 | 4.85969 |
| NFKBIZ | 4.45357 | 4.98707 |
| PMAIP1 | 4.42366 | 4.81869 |
| ZC3HAV1 | 4.23174 | 4.42906 |
| LIF | 4.22266 | 4.50932 |
| HERC5 | 4.08573 | 5.54689 |
| CYR61 | 3.99167 | 4.22102 |
| CCL2 | 3.86429 | 3.93527 |
| SOD2 | 3.70369 | 3.82299 |
| TNFAIP2 | 3.66225 | 3.90067 |
| - | 3.63644 | 4.4452 |
| BIRC3 | 3.53348 | 4.01871 |
| NGFR | 3.52317 | 5.08581 |
| - | 3.38992 | 4.20468 |
| NFKBIA | 3.33643 | 3.50711 |
| IRAK2 | 3.31163 | 4.66395 |
| ZC3H12A | 3.27735 | 3.71589 |
| ADAP1 | 3.23551 | 4.15319 |
| GCH1 | 3.20121 | 3.96548 |
| IFIH1 | 3.19997 | 3.63275 |
| VCAM1 | 3.18775 | 3.29171 |
| C11orf96 | 3.16278 | 3.88895 |
| SLC39A14 | 3.14406 | 3.42599 |
| ICAM1 | 3.03121 | 4.25487 |
| BBC3 | 3.02557 | 3.63885 |
| CCL8 | 2.99418 | 4.49302 |
| NCOA7 | 2.96432 | 3.27236 |
| ARRDC3 | 2.9586 | 3.23649 |
| NINJ1 | 2.91395 | 3.12006 |
| ZSWIM4 | 2.87246 | 4.14383 |
| CTGF | 2.83496 | 3.1797 |
| CCL5 | 2.81497 | 4.51378 |
| CH25H | 2.81312 | 4.08725 |
| XBP1 | 2.78117 | 2.90724 |
| RCAN1 | 2.72576 | 2.93171 |
| IFI44 | 2.70504 | 2.89401 |
| SAMD4A | 2.68482 | 3.03895 |
| DDX58 | 2.63703 | 3.08786 |
| BDKRB2 | 2.62678 | 2.94483 |
| HBEGF | 2.62382 | 4.0076 |
| MX1 | 2.61877 | 3.06641 |
| USP18 | 2.60167 | 2.87072 |
| TBX2 | 2.5914 | 3.61332 |
| PPP1R15A | 2.56902 | 2.91359 |
| NAMPT | 2.54539 | 2.80735 |
| ITPRIP | 2.54049 | 3.05113 |
| ZNF844 | 2.53784 | 4.14937 |
| FST | 2.53486 | 3.18341 |
| ISG20 | 2.46011 | 3.91588 |
| SLC16A6 | 2.44231 | 4.16586 |
| IL24 | 2.43888 | 3.96946 |
| IL12A | 2.43634 | 2.90777 |
| ZNF496 | 2.3697 | 4.09875 |
| SAMHD1 | 2.36764 | 2.96382 |
| TNFSF13B | 2.3619 | 3.66062 |
| RELB | 2.35028 | 2.84159 |
| IL15RA | 2.34336 | 2.71766 |
| MT2A | 2.3393 | 2.42211 |
| CCRN4L | 2.33243 | 2.95697 |
| DKK1 | 2.3127 | 3.85211 |
| ARL5B | 2.31247 | 3.21196 |
| STK40 | 2.3086 | 2.68371 |
| ERRFI1 | 2.29486 | 2.62856 |
| NAV3 | 2.28595 | 2.76588 |
| OAS1 | 2.26747 | 2.69177 |
| IRF7 | 2.26608 | 2.5556 |
| EDN1 | 2.26062 | 3.52405 |
| MFSD2A | 2.23523 | 3.70643 |
| SAMD9 | 2.22044 | 2.55031 |
| GBP5 | 2.21754 | 3.26102 |
| IFNGR2 | 2.2161 | 2.67084 |
| GFPT2 | 2.20374 | 2.44246 |
| CMPK2 | 2.2025 | 2.60218 |
| EGR1 | 2.18873 | 2.55899 |
| ZCCHC2 | 2.18021 | 2.64338 |
| SP110 | 2.17226 | 2.61797 |
| IFIT5 | 2.17105 | 2.43058 |
| TRIP10 | 2.16847 | 2.5715 |
| IRF1 | 2.16287 | 2.47451 |
| GADD45B | 2.15906 | 2.49302 |
| ZNF44 | 2.1507 | 2.91383 |
| EIF2C2 | 2.13311 | 3.18216 |
| PDCD1LG2 | 2.11126 | 2.87635 |
| FAM46A | 2.08917 | 2.36334 |
| STAT5A | 2.08704 | 2.5691 |
| GBP2 | 2.07948 | 2.34332 |
| C1QTNF1 | 2.07544 | 2.29394 |
| PTPN12 | 2.06118 | 2.32739 |
| HIVEP2 | 2.0602 | 2.71641 |
| ADPRHL2 | 2.05242 | 2.34361 |
| RIPK2 | 2.05072 | 2.34107 |
| ZNF20 | 2.03703 | 2.84778 |
| C8orf4 | 2.03672 | 2.22948 |
| PML | 2.03608 | 2.29317 |
| EPSTI1 | 2.01932 | 2.53122 |
| NEAT1 | 2.00818 | 2.10112 |
| TNFAIP6 | 2.006 | 3.06398 |
| JHDM1D | 1.99092 | 3.27324 |
| IFI44L | 1.96881 | 2.26929 |
| OTUD4 | 1.95892 | 2.34961 |
| FAM65B | 1.95477 | 2.87635 |
| SPSB1 | 1.94448 | 2.24847 |
| SPHK1 | 1.92855 | 2.37231 |
| PSMA2 | 1.91992 | 2.05252 |
| DUSP8 | 1.91544 | 3.57679 |
| C21orf91 | 1.90988 | 2.35748 |
| NFKBIE | 1.90978 | 2.40687 |
| TRIM26 | 1.90855 | 2.11973 |
| DUSP1 | 1.89925 | 2.62638 |
| TP63 | 1.89802 | 3.22999 |
| HERC6 | 1.87093 | 2.47071 |
| RHOC | 1.86074 | 2.01487 |
| WTAP | 1.8505 | 2.01587 |
| SMAD7 | 1.83553 | 2.27033 |
| CDKN2B | 1.83152 | 2.31058 |
| FMO5 | 1.81959 | 2.75082 |
| OAS2 | 1.81255 | 2.14731 |
| MDM2 | 1.80658 | 1.83134 |
| SPRY2 | 1.80468 | 2.52272 |
| PARP14 | 1.80447 | 2.14346 |
| JUN | 1.80417 | 2.00796 |
| PRELP | 1.80038 | 2.58262 |
| GDF15 | 1.79672 | 2.1415 |
| SLC2A3 | 1.7942 | 2.29139 |
| EHD1 | 1.79407 | 2.03746 |
| ZNFX1 | 1.77718 | 2.04882 |
| FTH1 | 1.77389 | 1.80685 |
| MAP3K8 | 1.76926 | 2.59625 |
| ID2 | 1.76754 | 1.91272 |
| STK17B | 1.76696 | 2.10042 |
| GADD45A | 1.75008 | 2.03332 |
| C17orf44 | 1.74697 | 2.46827 |
| IRF2 | 1.74441 | 2.01546 |
| CCNL1 | 1.73489 | 2.06426 |
| NFKB1 | 1.73398 | 1.98977 |
| ZNF267 | 1.7176 | 2.14263 |
| KLF4 | 1.697 | 2.25577 |
| ARRDC2 | 1.69321 | 2.24017 |
| IL11 | 1.69199 | 2.87635 |
| YPEL2 | 1.69199 | 2.87635 |
| UBXN7 | 1.68819 | 2.28134 |
| F3 | 1.6773 | 1.9556 |
| JAK2 | 1.67592 | 2.14605 |
| TGIF1 | 1.6751 | 1.99344 |
| DCP1A | 1.67 | 2.19623 |
| TIPARP | 1.66899 | 1.96075 |
| GBP3 | 1.66653 | 1.87083 |
| HERPUD1 | 1.66212 | 1.93585 |
| ZNF121 | 1.65766 | 3.25487 |
| ADORA2A | 1.65041 | 3.00411 |
| MCM7 | 1.64904 | 1.76064 |
| RHEBL1 | 1.64681 | 3.33579 |
| CLK1 | 1.64411 | 1.8497 |
| MAP2K3 | 1.64066 | 2.26867 |
| RPS8 | 1.63904 | 1.69573 |
| STK10 | 1.63731 | 2.30422 |
| PIM1 | 1.62938 | 1.89794 |
| SLC25A37 | 1.62702 | 2.09208 |
| MX2 | 1.61898 | 2.04772 |
| FLOT1 | 1.61727 | 1.71867 |
| MCL1 | 1.61272 | 1.84146 |
| NNMT | 1.61143 | 1.73139 |
| KYNU | 1.61005 | 1.6929 |
| LAMC2 | 1.60906 | 2.92366 |
| MIR100HG | 1.60861 | 2.13768 |
| VEGFC | 1.60558 | 1.81134 |
| NFAT5 | 1.59741 | 2.00236 |
| ZBTB43 | 1.59697 | 2.07408 |
| XAF1 | 1.59088 | 2.40687 |
| LAP3 | 1.58537 | 1.71712 |
| PNPT1 | 1.57138 | 1.85026 |
| FAM176A | 1.56191 | 1.8731 |
| ZNF134 | 1.55733 | 2.12933 |
| BCAR3 | 1.55009 | 1.9232 |
| SLC25A28 | 1.54332 | 1.89748 |
| RAB30 | 1.53888 | 3.35028 |
| ZNF442 | 1.53888 | 3.35028 |
| CDT1 | 1.53734 | 1.86547 |
| PLK2 | 1.53092 | 1.70059 |
| OGFR | 1.52142 | 1.8573 |
| ADM | 1.51753 | 1.8205 |
| SPAG1 | 1.51353 | 2.7146 |
| SLC38A2 | 1.51347 | 1.7991 |
| CLIC4 | 1.50587 | 1.65795 |
| RNF152 | 1.50504 | 2.07339 |
| STC1 | 1.49653 | 1.7014 |
| PLK3 | 1.4888 | 2.27324 |
| CXCR7 | 1.48852 | 2.29139 |
| E2F7 | 1.48584 | 1.75945 |
| ARHGAP31 | 1.48481 | 1.93029 |
| FGF2 | 1.48284 | 1.87123 |
| LGALS17A | 1.4821 | 3.09875 |
| PLA2G4A | 1.4626 | 1.64048 |
| APOL2 | 1.45948 | 1.80657 |
| DDX39A | 1.45448 | 1.65698 |
| DBT | 1.44481 | 1.94466 |
| OLFM2 | 1.44453 | 2.27121 |
| CLCF1 | 1.44426 | 1.9713 |
| BPGM | 1.44408 | 1.7776 |
| TRAF3 | 1.44102 | 1.97322 |
| MYC | 1.43177 | 1.94988 |
| IL1B | 1.42923 | 2.9103 |
| SLC22A23 | 1.42668 | 1.8633 |
| DENND5A | 1.42665 | 1.69672 |
| ETV7 | 1.4258 | 2.23329 |
| FUCA2 | 1.423 | 1.57719 |
| IKZF5 | 1.42227 | 2.09162 |
| PDPN | 1.42071 | 1.62971 |
| MDGA1 | 1.42008 | 2.84598 |
| GBP4 | 1.41809 | 1.83471 |
| TRIP12 | 1.41801 | 1.65575 |
| NSA2 | 1.41133 | 1.56058 |
| TRIM22 | 1.41028 | 1.62649 |
| C7orf41 | 1.41008 | 1.85589 |
| ZNF92 | 1.40801 | 2.19828 |
| MAFF | 1.40087 | 3.22999 |
| CLIP2 | 1.39853 | 1.75194 |
| NAB1 | 1.39598 | 2.05693 |
| MBIP | 1.392 | 1.63433 |
| TOP1 | 1.39145 | 2.06277 |
| STX3 | 1.38829 | 1.96946 |
| IGFBP4 | 1.37057 | 1.51459 |
| MANF | 1.37021 | 1.74825 |
| ATP5L | 1.3689 | 1.46796 |
| HES1 | 1.3677 | 1.76659 |
| CSRNP1 | 1.36555 | 2.39831 |
| RPS6KA3 | 1.36523 | 1.59277 |
| UXS1 | 1.35555 | 1.63557 |
| CCDC9 | 1.34924 | 1.87635 |
| SERPINE2 | 1.34904 | 1.44681 |
| HBP1 | 1.34818 | 1.79389 |
| ZNF823 | 1.34396 | 2.01837 |
| PSMD7 | 1.33925 | 1.5193 |
| ENC1 | 1.33844 | 1.74485 |
| MOBKL1B | 1.335 | 1.7902 |
| RPL10 | 1.32688 | 1.39365 |
| ZC3H12C | 1.3224 | 1.86609 |
| CCNG2 | 1.3179 | 1.55399 |
| WARS | 1.31706 | 1.43748 |
| SLC11A2 | 1.31552 | 1.9454 |
| DNHD1 | 1.31287 | 2.75082 |
| RPL37 | 1.30751 | 1.36072 |
| MED24 | 1.30543 | 1.58025 |
| VMP1 | 1.30418 | 1.49842 |
| PANK2 | 1.29956 | 1.62546 |
| COX5A | 1.29136 | 1.42769 |
| CEBPB | 1.29028 | 1.49503 |
| PPIF | 1.28457 | 1.44609 |
| EIF2AK2 | 1.28264 | 1.83392 |
| XRN1 | 1.27788 | 1.62481 |
| RPF1 | 1.27456 | 1.68371 |
| USP42 | 1.27386 | 1.58548 |
| BTG3 | 1.27207 | 1.5479 |
| PTPRE | 1.26978 | 2.13467 |
| FAM126A | 1.26698 | 1.45558 |
| MED25 | 1.26686 | 1.50552 |
| PANX1 | 1.26269 | 1.54278 |
| JARID2 | 1.25804 | 1.76742 |
| RAB5C | 1.25685 | 1.42586 |
| BATF2 | 1.25455 | 2.99183 |
| IL4I1 | 1.25455 | 2.99183 |
| ETS1 | 1.251 | 1.45744 |
| CWC22 | 1.25054 | 1.72602 |
| CSF1 | 1.25049 | 1.87635 |
| FLT3LG | 1.23992 | 1.46082 |
| C1orf77 | 1.23429 | 1.53401 |
| MEG3 | 1.23365 | 1.57343 |
| DNAJB6 | 1.23211 | 1.38177 |
| LMTK2 | 1.22816 | 2.87635 |
| NMI | 1.22264 | 1.67142 |
| CD74 | 1.2202 | 1.65295 |
| EBNA1BP2 | 1.21942 | 1.38872 |
| CTNNBL1 | 1.21919 | 1.61635 |
| MED30 | 1.21702 | 2.08657 |
| CAB39 | 1.21608 | 1.56805 |
| C1orf38 | 1.21478 | 2.26338 |
| UBE2L6 | 1.21076 | 1.36756 |
| ZNF433 | 1.20678 | 1.86125 |
| MTF1 | 1.20441 | 1.59837 |
| PIGF | 1.20246 | 1.87635 |
| FAM18B1 | 1.20177 | 2.13939 |
| RPL27A | 1.19677 | 1.27208 |
| XRCC6 | 1.19627 | 1.30811 |
| PDXK | 1.19572 | 1.49267 |
| CDK11A | 1.19509 | 1.47938 |
| PPA1 | 1.19412 | 1.34629 |
| DHX9 | 1.19017 | 1.42843 |
| CRELD2 | 1.18594 | 1.44617 |
| USPL1 | 1.18576 | 1.80797 |
| KIN | 1.18324 | 1.58801 |
| RRN3 | 1.18291 | 1.51525 |
| CASP4 | 1.1802 | 1.47454 |
| RNF121 | 1.17955 | 1.72108 |
| LAMTOR1 | 1.176 | 1.42145 |
| SKIL | 1.17383 | 1.96516 |
| SLC43A3 | 1.17253 | 1.44273 |
| CEBPD | 1.17056 | 1.27456 |
| HDAC9 | 1.16959 | 1.99766 |
| RPS25 | 1.16886 | 1.27759 |
| APOL6 | 1.16865 | 1.38122 |
| MKI67IP | 1.16807 | 1.6059 |
| HMGA2 | 1.166 | 3.02836 |
| RNF114 | 1.1615 | 1.28481 |
| EEF2 | 1.15726 | 1.21607 |
| CHMP4B | 1.15034 | 1.37287 |
| DUSP6 | 1.14983 | 2.20566 |
